# Supplementary material for: WordCommentsAnalyzer: A windows software tool for qualitative research
Source: F1000Res. 2018 Sep 4;7:536. Originally published 2018 May 3. [Version 2] doi: 10.12688/f1000research.14819.2 (PMC6137411; doi:10.12688/f1000research.14819.2)
Supplement: Supplementary file 3 [file f1000research-7-17614-s0002.tgz › b2a79496-4ada-47c4-9682-a99ce867836d.zip!/Eight Iranian Health Journals/Archives of Rehabilitation_14.docx]

© Archives of Rehabilitation. This work is licensed under CC BY-NC 4.0 (https://creativecommons.org/licenses/by-nc/4.0/).

Volume & Issue: دوره 15 - شماره 4

Title: سرمقاله: نگرش دانش آموزان دختر دبیرستانی به معلولیت جسمی

http://rehabilitationj.uswr.ac.ir/article-1-1273-fa.html

پدیده معلولیت سابقه ای به قدمت تاریخ بشری دارد و همچنان که تحقیقات باستان شناسان نشان می دهد، ناهنجاری های اسکلتی از عهدهای بسیار دیرین وجود داشته است. در حال حاضر بر طبق آمارهای موجود حدود 13 میلیون معلول با معلولیت های مختلف جسمی و ذهنی در کشور وجود دارد که با توجه به جمعیت کل کشور، تعداد بالایی را شامل می شوند . معلولیت جدای از محدودیت ها و مشکلاتی که از نظر کارکردی برای فرد معلول ایجاد می کند، سبب تأثیرات سوء روان شناختی در اثر تعاملات اجتماعی بر شخصیت افراد معلول شده که در این بین نگرش افراد جامعه به معلولیت نقش تعیین کننده ای دارد

Title: اثربخشی آموزش بازداری پاسخ بر حافظه فعال دانش‌‌آموزان با اختلال نارسایی توجه/بیش‌فعالی

http://rehabilitationj.uswr.ac.ir/article-1-1484-fa.html

هدف: پژوهش حاضر با هدف تعیین اثربخشی آموزش بازداری پاسخ بر حافظه فعال دانش‌‌آموزان پسر با اختلال نارسایی توجه/ بیش‌فعالی انجام شد. روش بررسی: پژوهش حاضر، یک مطالعه شبه آزمایشی با طرح پیش آزمون- پس آزمون و گروه کنترل بود. 40 پسر با اختلال نارسایی توجه/بیش‌فعالی 8 تا 10 ساله با روش نمونه‌گیری در دسترس از مدارس از مدارس شهرستان ورامین و قرچک انتخاب و به طور مساوی در یکی از دو گروه آزمایش و گروه کنترل (هر گروه 20 نفر) جایگزین شدند. گروه آزمایشی، در 10 جلسه آموزش (هفته‌ای 2 جلسه؛ هر جلسه 60 دقیقه‌) شرکت کردند در حالی که گروه کنترل در این جلسات شرکت نکرد. دانش‌آموزان با استفاده از آزمون حافظه فعال کورنولدی مورد ارزیابی قرار گرفتند. داده‌ها با استفاده از تحلیل کوواریانس با به کارگیری نرم‌افزار SPSS نسخه 16 تحلیل شدند. یافته‌ها: یافته‌ها نشان داد که آموزش بازداری پاسخ بر حافظه فعال دانش‌آموزان با اختلال نارسایی توجه/بیش‌فعالی اثر مثبت و معناداری داشته است (P>0/001). نتیجه‌گیری: نتایج حاکی از آن بود که برنامه آموزشی بازداری پاسخ سبب بهبود حافظه فعال دانش‌آموزان پسر با اختلال نارسایی توجه/بیش‌فعالی شده است. پیشنهاد می‌شود که متخصصان با طراحی و اجرای برنامه‌ آموزشی بازداری پاسخ، حافظه فعال دانش‌آموزان با اختلال نارسایی توجه/بیش‌فعالی را ارتقاء بخشند.

Title: مقایسه تاثیر تمرینات سنتی و تمرینات با حمایت وزن (BWST) بر عملکرد حسی حرکتی، کیفیت و کمیت راه رفتن در افراد ضایعه نخاعی پاراپلژی

http://rehabilitationj.uswr.ac.ir/article-1-1371-fa.html

هدف: هدف از این پژوهش مقایسه تاثیر تمرینات سنتی و تمرینات با حمایت وزن (BWSTT) بر عملکرد حسی- حرکتی در افراد پاراپلژی می باشد. روش بررسی:  تعداد 17 نفر ضایعه نخاعی پاراپلژی (ASIA B,C)، سن 793/1±53/32 سال، قد 658/1±71/175 سانتی‌متر، وزن 442/2±59/71 کیلوگرم و شاخص توده بدنی (BMI) 828/0±18/23 کیلوگرم بر متر مربع بطور دسترس و داوطلبانه به عنوان آزمودنی انتخاب شدند. این آزمودنی ها بر اساس نمره حسی و حرکتی به گروه تمرین BWSTT (10 = N) و گروه تمرین سنتی (7 = N) تقسیم شدند. آزمودنی ها در یک دوره 12 هفته‌ای، 4 جلسه 60 دقیقه ای در هفته شرکت کردند. تمرینات BWST شامل 15 دقیقه‌ گرم کردن روی دوچرخه ثابت و سپس 45 دقیقه تمرین با 50% وزن بدن روی دستگاه نوارگردان و در آخر 10 دقیقه تمرینات سرد کردن بود و در هر هفته 10% به وزن تحمل شده اضافه می شد. تمرینات سنتی شامل یک زمان 15 دقیقه ای گرم کردن روی دوچرخه ثابت و 45 دقیقه تمرینات کششی حس عمقی، انجام حرکات در دامنه حرکتی کامل و 10 دقیقه سرد کردن بود. یافته ها: داده ها نشان داد که تفاوت معنی داری در تغغیرات عملکرد حسی (P=0/002) و حسی (P=0/002)، عملکرد حرکتی (P=0/000)، آزمون شاخص راه رفتن (P=0/002)، آزمون راه رفتن 6 دقیقه ای (P=0/001) و 10 متر (P=0/001) بین گروه حمایت وزن (BWSTT) و گروه تمرینات سنتی وجود داشت. نتیجه گیری: تمرینات BWSTT در مقایسه با تمرینات سنتی و رایج می تواند در روند بهبود حس و حرکت و نیز کمیت و کیفیت راه رفتن افراد پاراپلژی موثر تر است.

Title: بررسی فضای آموزشی مدارس با توجه به ورود دانش‌آموزان دارای نیازهای ویژه به مدارس عادی

http://rehabilitationj.uswr.ac.ir/article-1-1474-fa.html

هدف: پژوهش حاضر بررسی فضای آموزشی کودکان دارای نیازهای آموزشی ویژه با توجه به ورود آنها به مدارس عادی بود. روش بررسی: در این پژوهش توصیفی از یک پرسشنامه محقق ساخته، مشتمل بر 6 حیطه و هر حیطه دارای تعدادی سؤال در زمینه مربوطه بود، پس از تایید روایی و پایایی مورد استفاده قرار گرفت. محتوای پرسشنامه را استانداردهای فضای آموزشی برگرفته از استانداردهای موجود در کشورهای آمریکا و ژاپن و همچنین استانداردهای موجود در سازمان آموزش و پرورش استثنایی ایران تشکیل داد. هر سوال از سه بخش مطابق استاندارد،خارج از استاندارد و وجود ندارد بود. یافته‌ها: نشان می‌دهد که مدارسی که به منظور حضور دانش آموزان با نیازهای ویژه مشخص گردید از استاندارهای فضای آموزشی کافی در خصوص ورودی وخروجی‌های مدرسه،شرایط فیزیکی کلاسها،پله‌ها، سطح شیبدار، سرویس بهداشتی و نهایتاً تدارکات ویژه برای دانش‌آموزان برخوردار نیستند. نتیجه‌گیری: به منظور تقویت طرح آموزش فراگیر می‌بایست مدارس به لحاظ فضای آموزشی مورد تجدید نظر قرار گیرند و در مدارس ساخته شده نیز بازنگری صورت پذیرد.

Title: نیازسنجی وسایل تکنولوژی کمک توانبخشی از نوجوانان دانش آموز کم شنوا – ناشنوا و والدین ایشان؛ شهر اهواز- سال 1392

http://rehabilitationj.uswr.ac.ir/article-1-1479-fa.html

هدف: هدف پژوهش، تخمین نیاز دانش آموزان نوجوان کم شنوا و ناشنوای شهر اهواز به وسایل تکنولوژی کمک توانبخشی بود. روش بررسی: وسایل کمک توانبخشی موجود برای افراد کم شنوا- نا شنوا بر اساس کاربرد در شش گروه طبقه بندی شد. از نمونه گیری طبقه بندی شده برای انتخاب 72 دانش آموز کم شنوا-ناشنوای مقاطع راهنمایی و دبیرستان، عادی و استثنایی و والدین اپشان استفاده شد..پس از کسب رضایت با دانش آموزان مصاحبه حضوری در مدرسه و با والدین مصاحبه تلفنی انجام شد: ابتدا از مشکلات دانش آموزان در شش عملکرد که برای آنها وسایل کمکی است، سؤال شد. آنگاه وسایل کمکی با ارائه تصویر توضیح داده شدند و از مشارکت کنندگان در مورد آگاهی از وجود هر وسپله، نیاز به آن و اولویت تأمین نیاز پرسیده شد. یافته‌ها: هر چند 69 نفر (96%) از سمعک استفاده می کردند؛ 44 نفر (61%) در استفاده از تلویزیون، 66 نفر (92%) در استفاده از ساعتهای زنگ دار ، 24 نفر (33%) در شنیدن صدای زنگ در و تلفن، 63 نفر (88%) در مکالمه تلفنی، 14 نفر (19%) در گفت و شنود حضوری، و 46 نفر (63/89%) در مشارکت در کلاس درس دچار محدودیت بودند. تقریباً هیچ یک از شرکت کنندگان وسایل کمکی شنوایی مورد اشاره را نمی شناختند. پس از آگاه سازی از وجود وسایل کمکی، اعلام نیاز ایشان به هر یک از انواع وسایل معرفی شده بین 2 نفر (3%) تا 69 نفر (96%) بود. نتیجه‌گیری: آگاهسازی گسترده نوجوانان دچار کم شنوایی-ناشنوایی از وجود وسایل تکنولوژی کمکی ضروری است. از یافته های حاصل از این پژوهش می توان در تدوین برنامه تأمین وسایل کمکی برای نوجوانان سود برد.

Title: ارزیابی مهارت های حرکتی ظریف و درشت و ارتباط آن با برخی شاخص های پیکرسنجی و عوامل محیطی در کودکان 3 تا 6 سال شمال شهر تهران

http://rehabilitationj.uswr.ac.ir/article-1-1483-fa.html

هدف: رشد مهارت های حرکتی ظریف و درشت یکی از موضوعات مهم در رشد مهارت های حرکتی بنیادی در سال های اولیه زندگی است. هدف پژوهش حاضر ارزیابی مهارت های حرکتی ظریف و درشت و بررسی ارتباط بین برخی شاخص های پیکرسنجی و عوامل محیطی با رشد مهارت های حرکتی ظریف و درشت در کودکان 3 تا 6 ساله حاضر در مهد کودک های شمال شهر تهران در سال 1392 بود. روش بررسی: جامعه مورد مطالعه مهدهای کودک منطقه 1 و 2 تهران بود که چهار مهدکودک به روش دردسترس انتخاب و به شکل داوطلبنامه تعداد 206 کودک دختر و پسر مورد مطالعه قرار گرفتد. اطلاعات از طریق پرسشنامه اطلاعات خانواده و آزمون رشد دنور-2 جمع آوری شد. یافته‌ها: به طور میانگین 56/06 درصد از کودکان در مهارت های درشت و 77/56 در مهارت های ظریف عملکرد طبیعی و پیشرفته داشتند. اما 23/86 درصد در مهارت های درشت و 14/9 درصد در مهارت های ظریف در معرض احتیاط، 12/83 درصد در مهارت های درشت و 7/56 درصد در مهارت های ظریف دچار تاخیر رشدی بودند. بین برخی از شاخص های پیکرسنجی مثل پهنای شانه، پهنای لگن، طول بازو، دور شکم و دور سینه با عملکرد برخی از مهارت های درشت حرکتی مثل پریدن، لی لی کردن، پرتاب کردن همبستگی های معنی دار وجود داشت (0/05>P). زمان مشاهده تلویزیون و مدت زمان خواب روزانه از جمله عوامل محیطی تاثیر گذار بر عملکرد شوت زدن با پا بود. نتیجه‌گیری: تعداد قابل توجهی از کودکان شرکت کننده در تحقیق حاضر در مهارت های حرکتی درشت ضعف داشتند، لذا تقویت و بهبود این مهارت ها نیاز به توجه و برنامه ریزی خاص دارد.

Title: تاثیر تغییر دامنه حرکتی مفصل مچ پا در KAFO فلزی بر تعادل در بیماران مبتلا به سندرم پس از پولیو

http://rehabilitationj.uswr.ac.ir/article-1-1421-fa.html

هدف: ارتوز Knee- Ankle Foot Orthosis (KAFO) متداولترین ارتوز مورد استفاده در افراد مبتلا به سندروم پس از پولیو (PPS ) می باشد. در ایران مرسوم ترین KAFO های مورد استفاده طرح های فلزی هستند که در عین مؤثر بودن مشکلاتی در هنگام استفاده از آنها گزارش شده است. ، هدف از مطالعه حاضر، بررسی تاثیر تغییر دامنه حرکتی مفصل مچ پا در KAFO فلزی بر تعادل در بیماران مبتلا به سندرم پس از پولیو می باشد. روش بررسی: در مجموع 22 فرد مبتلا به PPS در گروه سنی 37 تا 50 سال از افراد مراجعه کننده به مرکز جامع توانبخشی هلال احمر در این مطالعه شرکت کردند. جمع آوری داده ها با استفاده از دستگاه نروکام (مدل 8.0.3 )،. با مدت زمان پیگیری 2 هفته برای هر فرد تحت 2 حالت پوشیدن KAFO متداول و KAFO با زاویه مچ پای افزایش یافته انجام شد. یافته‌ها: میزان حداکثر جابجایی مرکز ثقل به سمت خلف (P<0/002)، میزان سرعت جابجایی به سمت خلف (P<0/018) هنگام پوشیدن KAFO با زاویه مچ پای افزایش یافته به طور معناداری بیشتر بود. میزان نوسان مرکز ثقل در زمان بلند شدن از روی صندلی هنگام پوشیدن KAFO با زاویه مچ پای افزایش یافته به طور معناداری کمتر بود (P<0/002). اما در سایر پارامترهای در نظر گرفته شده تفاوت معناداری مشاهده نشد. نتیجه‌گیری: بر طبق نتایج تغییر زاویه مفصل مچ پای ارتوزی در KAFO باعث بهبود برخی از شاخص های مرتبط با ثبات پاسچرال در افراد مبتلا به PPS می شود.

Title: بررسی تأثیر کفی پیش ساخته با دیواره بلند داخلی بر تغییرات راستای قدامی و خلفی پای دختران دچار صافی انعطاف پذیر کف پا

http://rehabilitationj.uswr.ac.ir/article-1-1502-fa.html

هدف: صافی کف پای انعطاف پذیر از شایع ترین بیماری های اندام تحتانی در بزرگسالان شمرده می شود که به دلیل قرارگرفتن ساختارهای پا در راستای نامناسب باعث درد و عوارض در مفاصل بالاتر و بافت نرم می شود . هدف از انجام این مطالعه بررسی تغییرات راستای پا در افراد دارای صافی کف پای انعطاف پذیرتحت عنوان پاسخ استاتیک پا به یک نوع کفی پیش ساخته می باشد. روش بررسی: مطالعه از نوع شبه تجربی بوده و جامعه در دسترس شامل 32 دختر مبتلا به صافی انعطاف پذیر با محدوده ی سنی 18تا28 سال بودندکه برای اندازه گیری تغییرات از دستگاه لیزراستفاده شده است .زاویه خلف پا با بررسی زاویه والگوس پاشنه و زاویه قدام پا با بررسی زاویه ساق و بخش جلوی پا،در حالت پای برهنه و با کفی دیواره بلند داخلی مورد ارزیابی قرار گرفته اند . یافته‌ها: نتایج نشان داد که استفاده از کفی سبب کاهش معنادار زاویه خط قدامی وخط خلفی می شود (P<0/001) . بطور میانگین باکفی دیواره بلند داخلی 5/3درجه کاهش در زاویه قدامی پاو 4درجه کاهش در زاویه پاشنه مشاهده شد (P<0/001). نتیجه‌گیری: این مطالعه نشان داد که کفی دیواره بلند داخلی می تواند راستای پا را به وضعیت نرمال نزدیک نماید . بدین معنا که انحراف بوجود آمده در راستای خلف و بخش جلوی پا را تصحیح کند . ضمنا خط NAS علاوه بر نشان دادن تغییرات راستای جلوی پا، حاکی از تاثیرارتز براین بخش بوده است .

Title: تأثیر حضور دیگران در تجربه‌ی زندگی افراد مبتلا به لکنت

http://rehabilitationj.uswr.ac.ir/article-1-1262-fa.html

هدف: بخش اعظمی از زندگی ما صرف تعامل گفتاری با دیگران می‌شود. بنابراین شکست و موفقیت در این روابط عمیقاً بر پیشرفت و تعالی ما به عنوان یک موجود اجتماعی تأثیر گذار است. از آن جا که پدیده‌ی ارتباط انسانی بسیار پیچیده و چند بعدی است، مطالعه‌ی آن در محیطی تجربی بسیار دشوار است. هدف از این مطالعه نشان دادن نقش حضور دیگران از دیدگاه افراد مبتلا به لکنت می‌باشد. روش بررسی: این پژوهش به شیوه‌ی کیفی و با رویکرد پدیدار شناسی انجام شد.۲۱ فرد بزرگسال مبتلا به لکنت گفتار به صورت هدفمند انتخاب شدند و به روش بحث گروهی متمرکز مورد مصاحبه قرار گرفتند. گفته های مصاحبه شوندگان در طول تمام جلسات ضبط شد و توصیف کلامی شرکت کنندگان به طور کامل نوشته شد. مضمون‌های حاصل از تجربه‌ی افراد دارای لکنت نسبت به زندگی توام با لکنت بدست آمد. در آخرین مرحله با استفاده از روش ون مانن، اطلاعات بدست آمده طبقه بندی گردید و مورد بررسی و تجزیه و تحلیل کیفی قرار گرفت. یافته‌ها: در بررسی حاضر، نقش دیگران در تجربه‌ی افراد مبتلا به لکنت در مضمونی به نام "حضور دیگران" قرار گرفت. از گفته های مشارکت کنندگان ۹ زیر مضمون پدیدار گشت که عبارتند از: ۱- احساس تحقیر شدن توسط دیگران، ۲-تلاش برای جلب رضایت دیگران در گفتگو، ۳-میزان شناخت دیگران از لکنت، ۴- جنسیت دیگران، ۵-مورد تمسخر قرار گرفتن توسط دیگران، ۶- ترحم دیگران، ۷- تکمیل صحبت توسط دیگران، ۸- کم تحملی و کم توجهی دیگران، ۹- موقعیت و مقام دیگران . نتیجه‌گیری: لکنت همراه با آشفتگی‌های گفتاری، مکث‌های هنگام برقراری ارتباط و رفتارهای تقلا گونه است که در افراد دارای لکنت تحت تأثیر طرف مقابل یا شرکای ارتباطی تشدید می شود. نحوه‌ی برخورد دیگران با فرد دارای لکنت بسیار مهم است. حضور دیگران در نگرش فرد مبتلا، به لکنت خود تأثیر به سزایی دارد که، در این میان آموزگاران، همشاگردی‌ها و اعضای خانواده بیشترین نقش را برخوردار می‌باشند.
